# Artificial intelligence and ultra-high performance computing methods and experiments for drug discovery: virtual screening, deep learning, molecular dynamics simulations, ADMET modelling, and experimental validation

**DOI:** 10.1186/s43556-026-00498-1

**Published:** 2026-09-03

**Authors:** Lunna Li, Lianna D. Soriano, Welela M. Kedir, Felix L. Hoch, Desmond K. Loke

**Affiliations:** 1 Department of Chemical Engineering, University College London, London, WC1E 7JE UK; 2 College of Letters and Science, University of California, Berkeley, CA 94720 USA; 3 Department of Science, Mathematics, and Technology, and, The AI Mega Centre, Singapore University of Technology and Design, Singapore, 487372 Singapore; 4 Faculty of Engineering, University of Southern Denmark, Odense, 5230 Denmark

**Keywords:** Chemical spaces, Deep learning, Molecular dynamics simulations, ADMET modelling, Experimental validation, Drug discovery

## Abstract

Recent years have witnessed considerable progress in computer-aided drug discovery, driven by the incorporation of computational technologies within both academic and pharmaceutical environments. This evolution is marked by a significant accumulation of data pertaining to detailed three-dimensional structural information, ligand properties, and their interactions with therapeutic targets. The augmentation of computational capabilities and the accessibility of extensive chemical libraries containing billions of drug-like small molecules have further facilitated this transition. To effectively utilize these resources, it is imperative to employ rapid computing methods for virtual screening, which encompass structure-driven in silico screening across vast molecular spaces, supported by efficient recurrent profiling techniques. Furthermore, advancements in deep learning methodologies are required to improve the accuracy of prediction concerning target functionalities and ligand characteristics, even when complete receptor structures are not available. Here, this review discusses the expansion of chemical space, advanced virtual screening, deep learning, molecular dynamics (MD) simulations, Absorption, Distribution, Metabolism, Excretion, and Toxicity (ADMET) modelling, and challenges for drug discovery. It examines how experimental validation integrates computational predictions with laboratory testing for effective candidate selection. Finally, it outlines future research directions for artificial intelligence-driven, ultra-high performance computing (UHPC) in drug discovery, offering new prospects for the economical creation of safer and more efficacious molecule-level therapies.

## Introduction

**Fig. 1:**
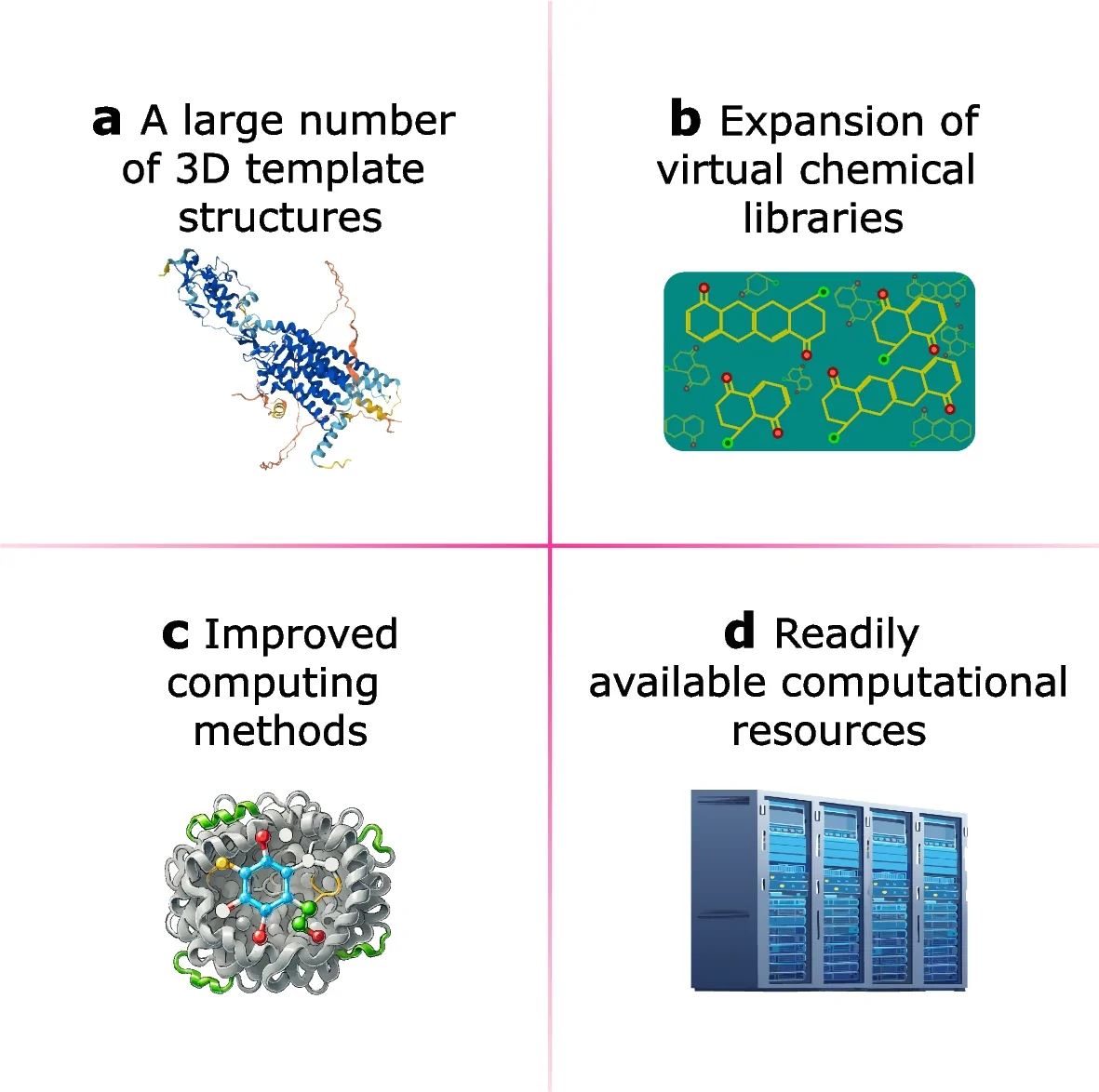
The advanced elements of virtual ligand screening (VLS) technology for the development of high-quality hits and leads. **a** Schematic diagram of diverse protein structures in the protein data bank. **b** A schematic illustration of extensive chemical libraries for screening and quick synthesis. **c** The schematic depiction of enhanced computational methods for virtual ligand screening, e.g., advanced methodologies for rapid flexible docking. **d** Schematic representation of accessible computational resources bolstered by the swift advancement of cost-effective cloud computing. The figure was created using Adobe Illustrator 2023

Drug discovery has transitioned from serendipitous observations to systematic, technology-driven processes, evolving from initial trial-and-error methods to high-throughput screening of vast chemical libraries [1–8]. The human genome mapping and reverse pharmacology enabled targeted screenings [9–11], whereas computer-aided drug design, molecular docking, and advances in virtual ligand screening (Fig. 1) enhance molecular structures [12–14]. Notwithstanding these advancements, the pharmaceutical industry faces a persistent productivity crisis, characterized by soaring research and development expenditures and protracted 15-year timelines that frequently do not correspond with new drug approvals [15, 16]. Traditional and preliminary computational models continue to grapple with the intricacies of human biology, leading to elevated failure rates for lead compounds in clinical trials due to unforeseen toxicity or insufficient efficacy [17–19].

Over the past five years, there has been a significant transformation, propelled by the integration of artificial intelligence and improved computational approaches into nearly all phases of drug discovery [20, 21]. Deep learning methodologies, e.g., Alphafold, have significantly transformed the field of protein structure prediction, facilitating the de novo synthesis of novel compounds [20–24]. Moreover, this period has witnessed the emergence of “lab-in-the-loop” platforms that enhance artificial intelligence predictions by integrating real-time information, thereby reducing development timelines by one to two years [25–27]. Additional significant advancements encompass the utilization of artificial intelligence in drug repositioning. Recent reviews in in the domain of drug discovery pertain to chemical spaces and computational methods [28, 29]; however, to most effectively generate new drugs in the future, exploit all of their unique therapeutic characteristics, and help drive their molecular design, they have to be connected to experimental validation. Previous reviews in this field have also mostly concentrated on a singular category of computational methods.

Herein, this review commences by discussing the expansion of chemical space enabled by advancements in both physical and virtual compound libraries. The artificial intelligence methods pertain to deep learning, while the ultra-high performance computing (UHPC) methods involve improved virtual screening, molecular dynamics (MD) simulations, and Absorption, Distribution, Metabolism, Excretion, and Toxicity (ADMET) modelling. It subsequently examines advanced virtual screening methods, e.g., synthon-based virtual screening methods, deep learning approaches, along with hybrid strategies that integrate physics-based and data-driven models. The review analyzes the application and constraints of molecular dynamics simulations, discusses ADMET modelling and its prediction difficulties, and highlights the importance of experimental validation. It concludes by summarizing challenges and future research directions for artificial intelligence-powered, ultra-high performance computing in drug discovery.

## The increase in the available chemical space

The limited diversity and size of screening libraries have hindered the identification of novel powerful ligands and the overall drug discovery process [30, 31]. A traditional high-throughput screening campaign utilizes libraries with 50,000 to 500,000 compounds, providing only a limited number of genuine hits after secondary validation [32–35]. These hits are non-selective, weak, and exhibit suboptimal pharmacokinetic and ADMET properties. Identifying these hits requires years of meticulous trial-and error optimization. Scaling high-throughput screening to several million compounds is only feasible for large pharmaceutical companies but does not significantly enhance the results’ quality [36–38]. Similarly, chemical libraries employing virtual screening were historically constrained to a repository of molecules obtainable from suppliers, typically comprising below ten million unique molecules, thereby rendering the scale benefit over high-throughput screening negligible.

**Fig. 2:**
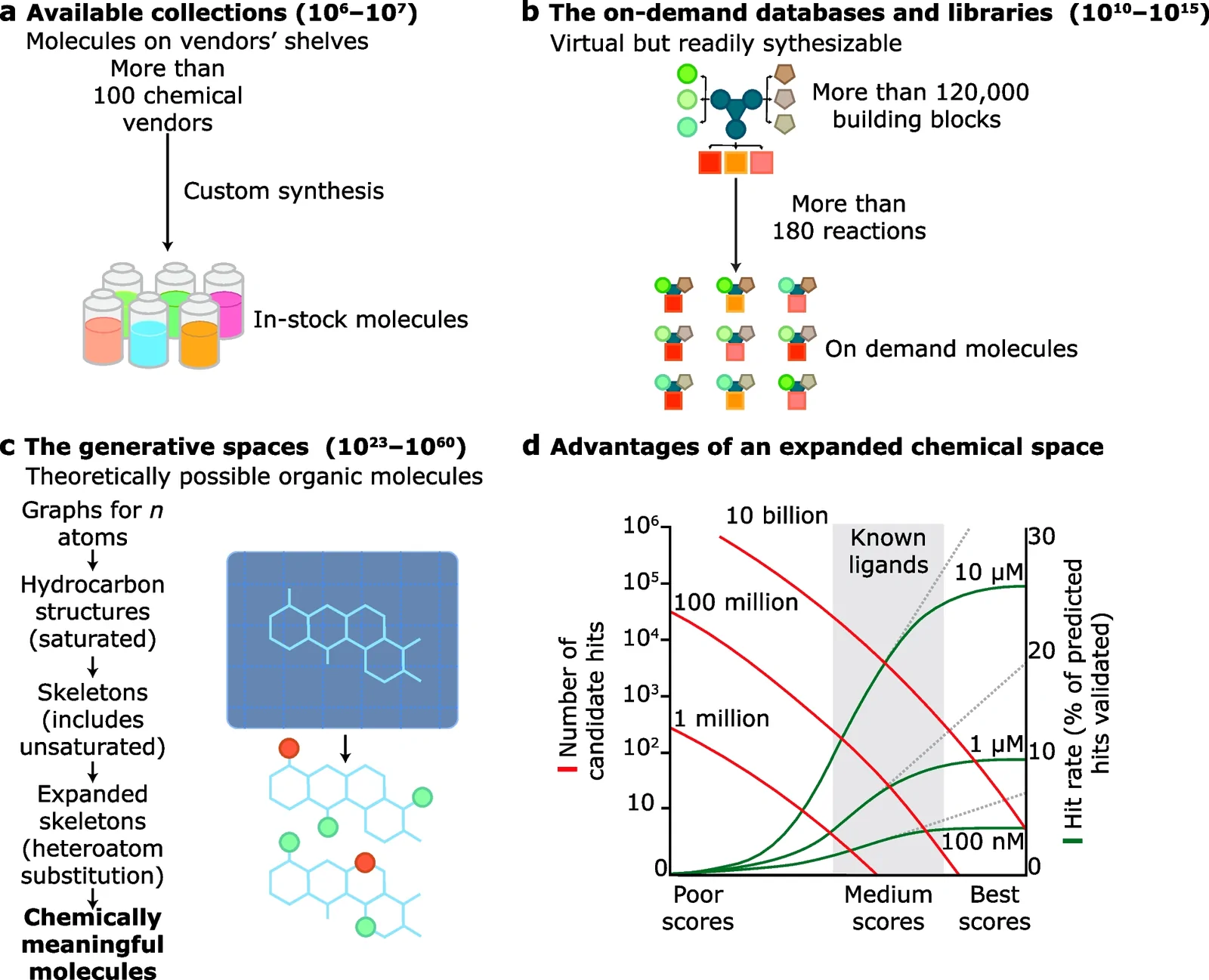
Comparison of chemical libraries in drug discovery and the advantages of augmenting chemical spaces for screening purposes. **a–c** Schematic illustrations of the **a** physical in-stock collections, **b** virtual on-demand databases, and **c** generative chemical spaces. **d** The distribution of initial hits with binding scores exceeding *X* for databases containing 1 million, 100 million, and 10 billion molecules (red curves), calculated from early virtual ligand screening and V-Synthes screening operations, and the connection between experimental hit rate and estimated docking score at 100 nM, 1 µM, and 10 µM levels (green curves). This partially quantitative analysis, varying by target, shows that screening over 100 million molecules address the constraints of smaller databases by broadening the hit distribution tail towards higher binding scores and elevated hit rates and identifying a greater proportion of experimental hits with enhanced affinity. The figure was created using Adobe Illustrator 2023

While pursuing comprehensive knowledge of the vast pharmaceutical-like molecular space, which is projected to exceed 10^60^ compounds, is an impractical endeavour, significantly augmenting the profiling of synthetically-accessible libraries to billions of previously unexamined drug-like compounds, whether virtual or physical (Fig. 2a–c), is anticipated to transform the drug development paradigm in various manners. It correspondingly augments the quantity of prospective active compounds in the preliminary profiling (Fig. 2d). Additionally, the extensive array of compounds in the database enhances the likelihood of identifying highly selective or potent compounds, along with those with excellent physiochemical features. Extensive in silico screening initiatives for multiple targets have demonstrated the discovery of high potency compounds with affinities frequently in the mid-nanomolar to sub-nanomolar level [35, 39, 40]. The availability of analogous hits in on-demand platforms also facilitates the establishment of significant structure–activity relationships (SAR) by cataloguing and subsequent optimization, hence minimizing the need for complex bespoke synthesis [41–43]. While the volume of the library is significant, well-designed gigascale libraries also enhance chemical variety, foster innovation, and increase patentability of the results, as nearly all synthetically-accessible compounds remained unsynthesized [44, 45].

### Libraries in physical form

Physical libraries have undergone innovative augmentation methodologies in high-throughput screening to increase their capacity limits. Recent advancements encompass combinatorial chemistry and the comprehensive aggregation of molecules for concurrent testing [42, 46]. Affinity-selection mass spectrometry is a prominent approach that facilitates the identification of binders from extensive pools of molecules without requiring labelling [47–49]. Moreover, DNA-encoded libraries (DELs) have been established, accompanied by economical techniques for their creation and screening, facilitating the control of about 10^10^ chemicals within a single test tube [50–52]. However, the DNA-encoded libraries and screening techniques have certain intrinsic limits. The traditional DNA-encoded libraries entail the tagging of ligands with distinct DNA sequences through linkers, so constraining the chemical variety permissible in the combinatorial assembly. Additionally, the screening techniques for conventional DNA-encoded libraries frequently result in a substantial incidence of false negatives due to the obstruction of critical binding moieties. Furthermore, a significant risk of false positives arises from the nonspecific attachment of DNA labels, necessitating costly off-DNA reconstruction to confirm the detected hit chemicals. To mitigate the difficulties inherent in the resynthesis process, it has been suggested that machine learning models, trained on the results of DNA-encoded library screens for particular targets, may be utilized to forecast pharmaceutical-like compounds from synthetically-accessible molecular spaces [50, 51, 53].

### On-demand virtual libraries

Virtual on-demand libraries signify a substantial progression in the pharmaceutical industry, employing computational methodologies to generate extensive chemical spaces [54–56]. Synthetic chemistry and cheminformatics have advanced to create these libraries, facilitating a more extensive investigation of molecules beyond conventional physical libraries. The REAL database by Enamine was the forerunner in 2017 [57–59], enabling expedited compound synthesis via optimized methods and a meticulously curated assortment of building blocks. The database expanded from around 160 million molecules to over 5.3 billion from 2017 to 2022, showing its effectiveness through successful screening programs that generated high-affinity ligands [60–63]. Recently, alternative commercial libraries, e.g., GalaXi, including approximately 8 billion chemicals, and CHEMriya, encompassing around 12 billion chemicals, have surfaced; however, their efficacy metrics are not publicly available [64, 65].

The modular design of on-demand virtual libraries also facilitates ongoing expansion; nevertheless, overseeing vast libraries with billions of compounds is unfeasible [66, 67]. Many companies utilize non-enumerated chemical regions defined by certain building blocks and reactions as a solution. For instance, the Pfizer Global Virtual Library (PGVL) employs a blend of proprietary chemicals and several processes to create an extensive virtual space. Alternate biopharmaceutical companies also maintain their unique virtual chemical spaces, although precise details may not always be readily accessible.

Theses expansive virtual spaces are also varied, innovative, and have minimal intersection, frequently under 10% [61, 68]. The Enamine’s REAL space, a prominent commercial library, maintains the synthesis speed and cost assurances of its original database, utilizing more than 170 reactions and a diverse selection of building blocks [68, 69]. Chemical techniques have advanced to encompass higher-order reactions, facilitating a possible expansion to 10^15^ molecules. Moreover, advanced cheminformatics tools have been created to navigate complex chemical spaces, enabling similarity searches and exploration of combinatorial potentials without exhaustive enumeration.

Alternate approaches to developing chemical spaces also entail generating theoretically synthesizable molecules grounded in principles of feasibility and stability. Examples encompass databases such as the Generated Databases (GDBs) [68, 69], which forecast compounds based on atomic quantities, and additional generative methodologies that utilize deep learning for ligand creation. However, the demonstrated efficacy of existing libraries, e.g., Enamine’s REAL space, contrasts with the ambiguous synthetic accessibility and success rates of newer spaces, prompting concerns regarding their practical usefulness in comparison to custom synthesis [51, 70–73].

## Virtual screening and deep learning approaches for drug discovery

After obtaining a comprehensive understanding of the growth of available chemical spaces, this section examines virtual screening and deep learning methodologies for drug development. Gigascale and terrascale chemical spaces, if maintained for drug variety and similarity, are anticipated to contain millions of possible active compounds and chemical series for every target, streamlining subsequent medicinal chemistry endeavours in pursuit of ultimate development candidates. Virtual libraries require innovative computational methods that meet specific demands for precision and rapidity. They have to exhibit sufficient speed to manage gigascale libraries, which take over 2,000 years to profile 11 billion molecules on a solitary central processing unit (CPU) core [68, 74]. Additionally, gigascale screening has to maintain excellent accuracy to prevent false-positive results, which undermine the scoring function. A one-in-million false positive rate in a 10^10^ chemical library results in 10,000 false hits, potentially overwhelming any candidate selection. Artefact characteristics and rate vary based on screening algorithms and the target, and have to be carefully considered throughout screening and post-screening. Cost-effective and practical strategies include the manual curation of the final compound list to identify atypical interactions, diversifying selections across various score ranges, choosing highly diverse hits, and selection informed by the consensus of two distinct scoring functions [75–77]. To optimize scoring efficacy in gigaspace, it is imperative to rectify as many outstanding defects in the scoring functions as feasible. By methodically finding and rectifying these deficiencies and reoptimizing the functions, researchers could attain greater selectivity within the score ranges where the highest true positives are generally located, resulting in more dependable outcomes [71, 72]. Additionally, validation in virtual screening is an essential, multiple-phase procedure aimed at verifying that computer models, e.g., docking or ligand-based models, precisely identify active compounds from an extensive library [78, 79], therefore reducing false positives and false negatives. This procedure guarantees the protocol’s dependability prior to its application in screening millions of chemicals, typically employing measurements, viz., root-mean square deviation (RMSD), enrichment factors, and receiver operating characteristic (ROC) curves. This section will examine emerging technologies and their optimal integration into the drug discovery and development pipeline to fully leverage expanding on-demand chemical spaces.

### Screening based on receptor structure

In silico screening is a method used in drug discovery that involves docking compounds from a chemical library onto a receptor configuration to determine its binding score. This strategy often utilizes a hybrid docking funnel, 3D shape-matching techniques, internal coordinate representation, or molecular mechanics [80, 81]. The focus is on ligand scoring systems that eliminate non-binders to reduce false-positive predictions. Blind evaluations of structure-based algorithm performance are conducted as part of the drug design data resource (D3R) grand challenge community initiative, demonstrating ongoing improvements in binding energy and ligand posture predictions for top algorithms [81, 82].

A multitude of effective structure-driven potential screening efforts has been demonstrated throughout the years, encompassing all significant categories of targets, with the latest emphasis on G protein-coupled receptors (GPCRs) [61, 68, 83, 84], while many different campaigns have been employed in the industry. The targeted candidate ligand sets identified through these screenings frequently exhibit advantageous success rates of 10–40% in experimental evaluations, resulting in unique active compounds for numerous targets with affinities ranging from 0.1 to 10 µM. Subsequent improvement of preliminary active compounds derived from routine screening databases containing below 11 million chemicals typically necessitates costly bespoke synthesis of analogues, a process that has only been feasible in a limited number of research studies.

The REAL space allows for the identification of hits within larger chemical spaces, resulting in a greater hit quality and quantity. This is particularly beneficial for challenging targets, e.g., the SARS-CoV-2 major protease (Mpro), which conventional virtual ligand screening has failed to achieve. Virtualflow of the REAL database successfully found active compounds within the 10–100 µM level, and these hits were then enhanced by rapid synthesis to create excellent leads [84, 85]. A separate ultra-large screen with 235 million compounds, using a novel configuration with a non-covalent antagonist, generated viable hits [85, 86]. Rapid optimization led to the identification of nanomolar Mpro inhibitors within four months using a blend of custom and on-demand chemistry [87–90]. The most effective molecule in the investigation revealed favourable in vitro ADMET characteristics, with cell-based antiviral potency of 78 nM and an affinity of 38 nM, akin to the clinically approved PF-07321332, i.e., nirmatrelvir [91, 92].

As library sizes grow, the docking’s computational time and expense become primary constraints in screening, despite the usage of parallel cloud computing. Virtualflow utilizes conventional stepwise filtration to screen over 1.4 billion Enamine’s REAL molecules, but this methodology increases computing expenses linearly with compound number and requires a fully enumerated library, rendering it less useful in rapidly evolving chemical domains [92, 93].

### Modular methods based on synthon

**Fig. 3:**
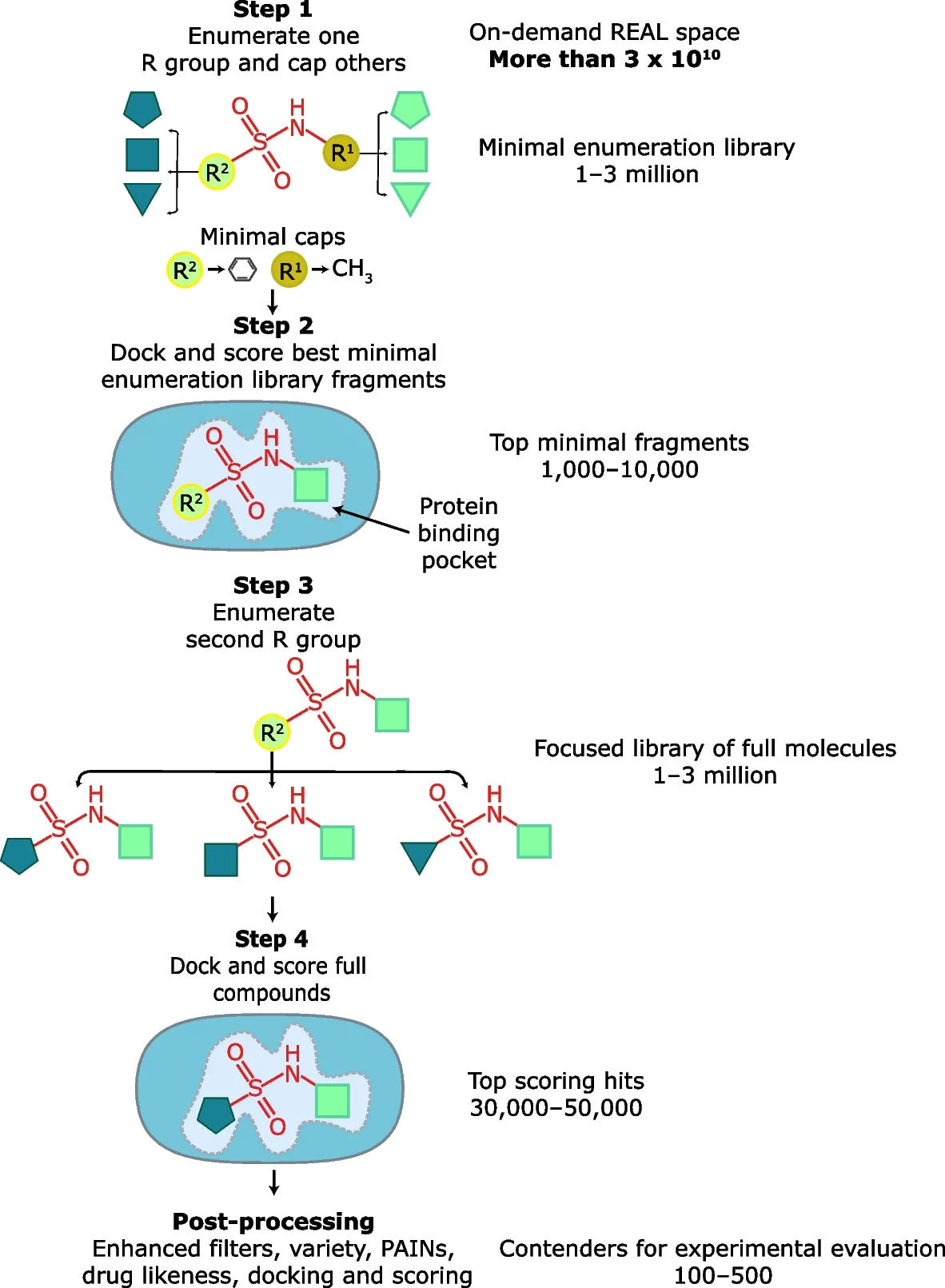
Hierarchical synthon screening. Schematic representation of the V-Synthes algorithm, which screens over 30 billion molecules in REAL space or bigger molecular spaces while enumerating and docking just small portions of molecules. The figure was created using Adobe Illustrator 2023

The virtual synthon hierarchical enumeration screening (V-Synthes) methodology is a new approach to drug discovery that uses fragment-driven techniques for synthetically-accessible molecular spaces (Fig. 3). The process begins by cataloguing REAL space processes and building elements, viz., synthons, and subsequently creates a small database of typical molecular fragments by thoroughly connecting synthons at a single attachment point while encapsulating the other locations with either a phenyl or methyl group [69, 94]. Docking-driven screening helps select the highest scoring fragments, which are then evaluated against the target pocket [95–97]. The leading 40,000 complete molecules from REAL space are subjected to advanced docking settings or techniques, and the best-performing lead compounds are evaluated for innovation, variety, and desirable pharmaceutical-like characteristics [98–101]. The optimal 50–500 compounds are chosen for synthesis and evaluation. The integration of synthons with structures in the V-Synthes algorithm, sealed with inert small ensembles, is essential for precise fragment forecasts, as reactive moieties of building elements and structures frequently generate robust interactions that are lacking in the complete molecule. Furthermore, the method assesses the fragment-bounded conformation within the target, favouring active compounds with minimum sealing oriented towards an area of the pocket that allows for fragment expansion [69, 102–104].

V-Synthes, a virtual ligand screening technique, has also shown an approximately 25% success rate for submicromolar ligands, consuming 100 times fewer computational resources and outperforming conventional screening by fivefold [105–107]. Moreover, it has identified one hit in the low nanomolar range in an investigation on ROCK1 kinase screening [69]. Furthermore, V-Synthes has been utilized for additional therapeutic targets with well-characterized pocket architectures [105, 106].

BioSolveIT has also created a comparable methodology, viz., chemical space docking (CSD), for two-component processes [108, 109]. This approach is more rapid, since it attaches separate building element pieces and subsequently connects them with structures and other synthons. Nevertheless, the increased speed entails trade-offs: the docking of miniature fragments without structures becomes less dependable, and their reactive moieties frequently exhibit dissimilar properties compared to the reaction result. This leads to significant drug-target interactions that are extraneous to the ultimate molecule and thus misdirect the fragment choosing process. This aspect is particularly applicable to cycloaddition processes and three-component structures, which require additional verification in chemical space docking.

Structure-based modular approaches also enhance potential hit quality, chemical diversity, and abundance by identifying ligands with significant chemical novelty. They uncover compounds that were not previously generated and do not rely on existing data, ensuring patentability for active compounds and chemical series from ultra-large virtual screening [86, 110]. Reliable analogues provide comprehensive structure–activity relationship analysis for optimal candidates, resulting in selectivity and potency enhancements. Multilayer readily-accessible chemical space enhancements, e.g., MAke on-DEmand (MADE) building elements, facilitate further lead optimization via “virtual Medchem”, minimizing the need for extensive bespoke synthesis [70, 110, 111].

### Quantitative structure–activity relationship methodologies

The quantitative structure–activity relationship (QSAR) modelling is an advanced method in ligand-based drug discovery, initially created to forecast the biological activities and/or characteristics of compounds [112]. A quantitative structure–activity relationship model employs a mathematical framework that delineates the correlation between biological activity and molecular attributes as *y* = *f*(*x*_1_, *x*_2_,…, *x*_n_). In this equation, *y* signifies the estimated biological activity or property of interest, whereas *x*_1_, *x*_2_, …, *x*_n_ represents the molecular characteristics, e.g., counts of atoms and bonds, the logarithm of the octanol–water partition coefficient (logP), and molar weight. The function *f* represents an empirically established mathematical correlation that pertains to these descriptors to ascertain property values for diverse compounds.

Conventional quantitative structure–activity relationship modelling employed fundamental regression techniques and concentrated on a restricted set of analogous compounds. Nevertheless, the domain has markedly progressed and utilized various enhanced methodologies to model and evaluate huge databases [113, 114]. Commonly recognized quantitative structure–activity relationship models include pharmacophore-based, local and global models. Recently, researchers have developed a ligand-based 3D quantitative structure–activity relationship model that evaluated antagonistic effects against the adenosine receptor 2 A (A_2A_) utilizing a collection of 268 unique chemicals [115–120]. The model was subsequently employed to assess 1897 different small molecules, underscoring the difficulties faced in safety screening due to novel chemistry. This work highlighted the essential analysis of pharmacophore-based 3D quantitative structure–activity relationship models related to safety assessments [118–120].

The primary objective of a quantitative structure–activity relationship model is to identify correlations between biological activity and molecular descriptor values, hence enhancing predictive accuracy [115, 121–123]. Reducing prediction error is essential, typically accomplished by measurements, e.g., the sum of squared differences between the projected and actual activities. Efficient data pre-processing and preparation are essential before developing these models and validating their accuracy, guaranteeing dependable results in biological activity predictions [115, 117, 124].

### Deep learning and data-driven methods

Artificial intelligence-driven deep learning methods are increasingly being utilized in drug discovery, e.g., target selection, lead optimization, and translational medicine, due to advancements in Alphafold, ChatGPT, and facial recognition [125, 126].

Data-driven methods in drug discovery have been widely used, including conventional machine learning methodologies, i.e., neural networks, support vector machines, and random forests. These methodologies predict ligand characteristics and on-target activity, but with variable outcomes [127–129]. Quantitative structure–property relationship models forecast pharmacokinetic and physiochemical properties, with extensive experimental datasets for model training [115, 116, 130–132]. Quantitative structure–activity relationship techniques focus on chemical scaffolds and specific targets, optimizing lead potency and affinity. Techniques such as network-based methodologies, chemical similarity clustering, and comprehensive ligand-target binding databases have been proposed for drug repurposing [115, 130].

Deep learning has revolutionized data-driven models, uncovering complex non-linear correlations and enabling the investigation of larger, diversified datasets. This has led to extensive literature on deep learning approaches and applications in drug discovery. Artificial intelligence requires extensive input datasets for predictive models, and quantitative structure–property relationship’s databases have been compiled to measure properties, e.g., in vitro substitutes for brain permeability and oral bioavailability, lipophilicity, and solubility, enabling the prediction of these properties in various compounds [133–136].

Quantitative structure–activity relationship models’ quality varies based on data availability, with the greatest advancements seen in aminergic G protein-coupled receptors and the kinase superfamily. The Illuminating the Druggable Genome (IDG-) DREAM drug-kinase binding prediction challenge has benchmarked the premier machine-learning quantitative structure–activity relationship models, e.g., deep learning-based algorithms, gradient boosting, and kernel learning [130, 132]. The highest-performing model used protein sequence identity, kernel regression, and affinity data from over 50,000 kinase-compound combinations, incorporating 527 kinases and 13,608 compounds obtained from public databases, e.g., ChEMBL and PubChem, for its training dataset. The conventional optimal deep learning model utilized over 900,000 experimental ligand-interaction data values for training, but underperformed compared to the more basic kernel model [137, 138]. The optimal models achieved a root-mean-square error of approximately 0.9 and a Spearman rank coefficient of around 0.5 for predicted compared to experimental pKd values, comparable to individual-point empirical testing for kinase suppression. This accuracy could be beneficial in screening for active compounds for underexplored kinases and directing molecule optimization. The kinase group is the largest class with over 500 targets, exhibiting significant cross-selectivity. The generalizability and efficacy of deep learning and machine learning techniques for these and additional targets have yet to be evaluated [139–141].

Artificial intelligence-driven drug discovery aims to create universally relevant representations of binding potencies based on information from ligand–protein 3D structures and known ligand activities. These models utilize various methodologies for network topologies and data representation, e.g., 3D deep convolutional neural networks, spatial graph-convolutional models, and their amalgamations [142, 143]. However, an investigation found that conventional explicit characterization of non-covalent molecular interactions in PDBbind compounds does not generate quantitative advantages over more basic estimations [143–146]. The effective performance of deep learning approaches utilizing PDBbind relies on the memorization of analogous ligands and receptors, in contrast to the assimilation of overarching information about their binding interactions. This may be due to the traditional PDBbind database’s inability to represent “negative space” ligands with unsatisfactory interaction patterns [147, 148].

The incident highlights the necessity for a more profound comprehension of deep learning algorithms and their reliance on training information. Research discloses that traditional models using restricted datasets overfit and misperform, leading to their classification influenced by subjective elements or as “ineffective”. Statistical instruments are being developed to validate the models’ efficacy, with a proposed approach being stability, computability, and predictability for “veridical data science” [149, 150]. The artificial intelligence community recognizes the need for quality data to bridge the “production gap”, where machine learning models fail in real-world applications. Efforts are being made to create tools that render artificial intelligence “explainable”, particularly in drug discovery applications.

Artificial intelligence is transforming drug discovery, with artificial intelligence-driven therapeutic candidates progressing to preclinical and clinical trials [151, 152]. These compounds are effective in vivo inhibitors of the receptor tyrosine kinase, viz., Discoidin Domain Receptor 1 (DDR1), a key player in fibrosis. Phase I clinical studies for ISM001-055, a drug for idiopathic pulmonary fibrosis, have been announced [151, 153]. A number of candidates targeting G protein-coupled receptors, e.g., those for the A_2A_, dual 5-HT_1A_–5-HT_2A_, and 5-HT_1A_ receptors, have progressed to clinical trials, a process significantly enhanced by the use of artificial intelligence. Artificial intelligence serves as a pivotal catalyst by enhancing hit discovery and lead optimization processes to forecast safer and more effective molecular structures, thereby expediting the progression from computational design to clinical stages [153, 154]. Moreover, artificial intelligence aids in multiple phases of G protein-coupled receptor drug discovery [155, 156]. In target identification, artificial intelligence examines biological data to pinpoint promising G protein-coupled receptor targets and elucidate illness causes. In hit discovery and virtual screening, artificial intelligence adeptly evaluates extensive chemical libraries to identify candidate compounds that interact with the target. Lead optimization employs artificial intelligence to forecast and enhance the qualities of drug candidates, e.g., ADMET characteristics, hence improving efficacy and minimizing side effects prior to preclinical testing. Moreover, artificial intelligence facilitates de novo drug creation by generating innovative chemical structures with certain attributes and assists in drug repurposing by pinpointing existing pharmaceuticals that may be useful against various ailments through targeting G protein-coupled receptor pathways. These success stories come from the G protein-coupled receptor and kinase families, with compounds resembling established high-affinity scaffolds. Future deep-learning drug prospects have to improve their uniqueness and application spectrum [153, 154, 157, 158].

### Computational methods that are hybrid in nature

Physics-derived and data-based methodologies offer unique benefits and constraints in forecasting ligand affinity. Structure-driven in silico docking generalize to three-dimensional targets, improving accuracy and reducing false positives. Conventional data-driven methods, which operates faster with general processing unit acceleration and substitute for structures, face challenges in generalizing beyond data-abundant target classes. Initiatives are underway to integrate these methodologies in synergy for drug discovery [157, 158].

The combination of physics-derived docking and data-based scoring systems in virtual screening methodologies can be advantageous, as it improves the findings’ quality and reduces false-positive rates. The drug-design data resource grand challenge 4 showed that methods integrating physics-derived and machine learning scoring surpassed those using non-machine learning approaches [159–161]. The Critical Assessment of Computational Hit-finding Experiments (CACHE) focuses on comprehensive benchmarking of these methodologies in the future, evaluating five scenarios pertinent to real-world lead generation and optimization [160–162].

Precise physics-derived docking results and empirical data can also be utilized to train 3D deep learning or generalized graph models to predict ligand-receptor affinity. This enhances the training dataset and equilibrates positive and negative binding instances, reducing overtraining concerns [100, 163, 164]. Deep learning-based 3D scoring systems have been developed, with Radial and Topological Convolutional Neural Network (RTCNN) being the most recent example, but their practical applicability is yet to be established [165, 166].

The use of artificial intelligence-generated models such as Rosettafold or Alphafold2 has also been explored to extend the application of structure-driven docking to targets without atomic-resolution structures. These models have shown utility in various applications, e.g., protein-peptide and protein–protein docking [167, 168]. They are anticipated to enhance the precision and extent of molecular modelling. An investigation found that Alphafold2 models, enhanced with additional artificial intelligence methodologies, helped identify a small-molecule antagonist with a moderate potency of approximately 9 µM. However, the overall benchmarking of traditional Alphafold2 models’ efficacy in virtual screening generated inconclusive results. In practical scenarios when structural similarities in the ligand-binding site are less apparent, prototypical Alphafold2 algorithms have exhibited subpar performance in small molecule docking for antibacterial and G protein-coupled receptor targets [75, 169]. Nevertheless, the Alphafill method facilitates the transfer of ligands and small-molecule cofactors from protein data bank structures to analogous Alphafold2 models, hence aiding in the validation and optimization of these models. Additional evaluation of their efficacy for docking and in silico screening is in progress [75, 170].

Conventional hybrid iterative methodologies, e.g., deep docking, active learning, and MolPal, have also been proposed to expedite the virtual screening of extensive chemical libraries. These methods utilize structure-based docking to train machine learning models, filtering the entire library to diminish its size. However, their scalability to rapidly expanding chemical spaces remains uncertain [74, 171, 172].

Traditional rapid docking algorithms and machine learning models also have limited accuracy in predicting binding potencies or affinities. To improve potency predictions, a condensed library of candidate binders can be analyzed and prioritized utilizing advanced physics-derived methodologies, viz., free energy perturbation techniques. General processing unit-accelerated calculations can be utilized in subsequent processing during in silico screening initiatives, enhancing success rates for strong-affinity candidates and lead optimization phases. This approach offers a wider use in subsequent processing during in silico screening initiatives [93, 161, 173].

## Molecular dynamics simulations in drug development

After acquiring a thorough knowledge of virtual screening and deep learning approaches, this section investigates molecular dynamics simulations in drug discovery. Molecular dynamics simulations are crucial computational methods in drug research, enabling timely prediction of atomic movement within molecular systems using physics-based intermolecular interactions [157, 158, 163, 174]. This methodology simplifies the complexity of wet lab assays and provides atomistic insights into biomolecular behaviours, rendering molecular dynamics simulations a computational microscope. They have been widely applied to elucidate the mechanisms of protein misfolding and aggregation-related diseases, and to identify molecular modulators. Recent studies suggest that molecular dynamics simulations could be utilized to identify therapeutic candidates for neurological disorder treatment or intervention [175, 176]. In the broader context of drug development, they provide valuable insights at multiple stages—from hit discovery to rational drug design [177–179].

Structure-driven drug development typically relies on molecular targets, e.g., proteins, derived from experimental techniques, viz., homology modelling, X-ray crystallography, or nuclear magnetic resonance (NMR), and databases such as the conventional AlphaFold protein structure database and protein data bank. Nevertheless, the static conformations of proteins from these sources may not accurately represent the biochemically relevant form, increasing the likelihood of false hits in in silico screening. To address this limitation, incorporating conformational dynamics in protein target has been advocated, for instance, through computational techniques such as molecular dynamics simulations, to enhance in silico screening and diminish false hits in the initial phases of drug development [102, 180]. Additionally, molecular dynamics simulations had been documented to generate several protein conformations that complement experimentally derived structures, such as those obtained from cryo-electron microscopy, nuclear magnetic resonance, and X-ray crystallography. These conformations can be subsequently used in ensemble docking to improve the identification of potential ligands [181–183]. This approach enhances virtual screening by revealing additional druggable sites and facilitating more accurate hit identification. For example, researchers have employed molecular dynamics-based approaches to identify novel aldoses reductase inhibitors (ARI) and alternative inhibitors, resulting in lead drugs that demonstrate significant biological activity and diminished off-target effects relative to the commercially available Epalrestat [184, 185]. Occasionally, protein targets acquired from traditional data repositories lack comprehensive active site information. A notable example includes recent studies of a 500-ns molecular dynamics simulation on renin, an important target for antihypertensive agents in the Renin-Angiotensin system for antihypertensive therapy [186–188]. The simulations showed binding pocket details absent in the static crystal structure obtained from the conventional protein data bank, highlighting molecular dynamics’ capacity to uncover cryptic binding sites that can be otherwise inaccessible in structural models [189, 190].

### Ensemble docking using molecular dynamics-derived states

Molecular dynamics simulations are utilized in the creation of structural ensembles owing to the extended duration of traditional all-atom molecular dynamics simulations. Biased molecular dynamics, which imparts external forces or modified potentials to accelerate structural sampling to help the system escape local energy minima more efficiently, have been utilized to address this problem. Conformations derived from molecular dynamics simulations exhibit excellent performance compared to conventional individual crystal structures and alternative ensemble generation techniques. However, identifying the optimal conformations remains elusive due to the extensive number of conformations generated in traditional molecular dynamics simulations [30, 191, 192]. Recent research employing Gaussian accelerated molecular dynamics simulations to generate protein conformations of the Adenosine A1 receptor (A1AR) protein for docking purposes found that ensembles derived from molecular dynamics outperformed individual cryo-electron microscopy structures [192–194]. Similar strategies have been applied to targets such as vascular endothelial growth factor receptor 2 (VEGFR2), cyclin-dependent kinase 2 (CDK2), anaplastic lymphoma kinase (ALK), and cathepsin B, demonstrating the broad applicability of molecular dynamics-based ensemble generation. Notably, G protein-coupled receptors, which possess multiple small-molecule binding sites, have benefited from molecular dynamics simulations to discern druggable states of membrane-associated G protein-coupled receptor structures [83, 84, 171]. The ability of molecular dynamics-derived ensembles to more diversely capture protein flexibility highlights their critical role in modern computer-driven drug design.

### Energetics evaluation

Molecular dynamics simulations offer stability indicators and energetic insights via trajectory simulations, utilizing methods, e.g., Molecular Mechanics-Generalized Born Surface Area (MM-GBSA) and Molecular Mechanics-Poisson-Boltzmann Surface Area (MM-PBSA), to determine binding free energy, which indicates the ligand’s affinity for the target [195–197]. Although alternative computational methods ascertain free energy, those based on molecular dynamics simulations exhibit enhanced efficacy and reduce false positives. Assessing binding free energy is essential in drug development, as a ligand’s therapeutic effectiveness depends on its binding affinity to the biological molecule. The umbrella sampling methodology emerged as an essential method in molecular dynamics simulations for evaluating the affinities of target-ligand complexes, with binding affinity determined as the disparity between the highest and lowest free energy variations utilizing the potential of mean force (PMF). Recent research on Alzheimer’s disease and Acetyl-cholinesterase (AchE) inhibitors underscores the significance of umbrella sampling for drug development [198–200]. The in silico investigation revealed 5-carboxy-2′-deoxyuridine as a potential drug contender against AchE, exhibiting binding energy of –11.4 kcal/mol as assessed by the potential of mean force, which accorded with the experimental binding affinity [195–197].

### Limitations of classical molecular dynamics simulations

Conventional molecular dynamics simulations are valuable across multiple stages of drug discovery, including lead optimization. However, their utility is limited by the requirement for long simulation times to generate statistically meaningful data [201, 202]. While a few nanoseconds may suffice to sample binding modes in simple protein–ligand systems, many biologically relevant processes occur on the microsecond to millisecond timescale—requiring simulations involving billions of time steps. As system complexity increases, the computational cost and time scale proportionally, posing significant challenges [201–205]. To mitigate these limitations, significant advancements have been made in computational hardware. Traditional supercomputers, viz., Anton, have been developed to address the limitations of simulation duration, but they remain inaccessible to most researchers. Alternate supercomputers with specialized hardware, e.g., Fastrun, DM Engine, and MDGrape, have been developed to improve computational efficiency [201, 206, 207]. Additionally, the emergence of advanced graphical processing units has made molecular dynamics simulations more cost-effective and accessible. Another approach is to construct a model system that incorporates prolonged time steps for the leap-frog integrator. All-atom molecular dynamics simulations typically exhibit a time step of 2 fs, but coarse-grained (CG) dynamics, where 2–5 heavy atoms are represented as a single pseudo atom, simulate systems with much extended time steps. The model had been utilized to investigate macromolecular targets, e.g., lipid membranes and proteins [195, 208, 209]. Coarse-grained modelling of proteins in conjunction with small-molecule ligands presents a significant challenge, requiring well-parameterized force fields and careful coarse-graining strategies to accurately capture molecular interactions [129, 208]. Recent advancements in the Martini force field utilizing open-source software, e.g., GROMACS and OpenMM—particularly its enhanced parametrization—have demonstrated excellent performance over all-atom molecular dynamics in the simulation of protein–ligand inhibitor systems within a coarse-grained framework. An increasingly popular approach for expediting molecular dynamics simulations by facilitating lengthened time steps in the leap-frog integrator is hydrogen-mass repartitioning (HMR). The hydrogen-mass repartitioning can be crucial in expediting the identification of prospective development candidates through membrane biophysics [126, 129].

## ADMET modelling and prediction

The preceding section has examined molecular dynamics simulations in drug development. This section explores ADMET modelling and projection. The therapeutic efficacy of a drug depends on its biological activity and the appropriate ADMET profile. In silico methodologies are crucial for assessing the pharmacokinetic efficacy of drug agents, minimizing the reliance on animal testing [103, 210]. Chemically active molecules adhere to specific parameters established by pharmacokinetic qualities to qualify as drug candidates. Preclinical candidates have to not exhibit toxicity, be excreted post-activity through the excretory system, undergo appropriate hepatic metabolism, demonstrate effective distribution, and have a propensity for absorption into the target surface [103, 210]. The Lipinski’s Rule of Five (Ro5) is a fundamental, though not obligatory, guideline in drug discovery that aims to predict the oral bioavailability of small-molecule pharmaceuticals. Although extensively employed to sift through extensive chemical databases [211–215], strict compliance is not required for a compound to qualify as a drug, as several effective medications violate one or more criteria. Lipinski’s guidelines for drug-likeness are based on specific physiochemical properties, e.g., partition coefficient, molecular weight, hydrogen bond donors and acceptors. Bioactive compounds, phytochemicals, and ligands that contravene numerous requirements are deemed to possess inadequate absorption [216–218]. This ensures that potential drugs meet the necessary criteria for effective drug discovery.

Various software applications have been created to forecast ADMET characteristics of drug candidates, e.g., Metasite, OSIRIS, and QIKprop [219]. Web servers, viz., admetSAR, ADMETlab, and SwissADME, are widely used for predicting ADMET properties in academic, industrial, and pharmaceutical contexts [216, 220, 221]. AdmetSAR 2.0 and Molinspiration are recognized online platforms for identifying physiochemical properties [216, 220, 221]. Early-stage prediction of drug-like and ADMET qualities of ligands using conventional web servers and in silico tools is crucial, but uncertainties exist regarding several aspects when evaluating substances based on their drug-like and pharmacokinetics characteristics [221–223].

### Risk of discarding valuable compounds

Researchers frequently exclude promising drug candidates due to their lack of drug-likeness and adverse ADMET properties. This may result in the elimination of lead candidates throughout the screening procedure [16, 224]. For instance, acarbose, a typical regulatory-approved small molecule for diabetes, is anticipated to exhibit inhibition of the human ether-à-go-go channel, oral toxicity, Ames mutagenicity, and hepatotoxicity. It contravenes Lipinski’s drug-likeness criteria by breaching 3 of the 4 criteria outlined in the rule of 5 [225, 226]. A computational researcher might exclude conventional acarbose, so potentially forfeiting a promising lead chemical for the treatment of type-2 diabetes. Although it is logical to exclude hit compounds with suboptimal ADMET profiles, researchers should take additional factors into account [227, 228]. The pharmacokinetics and drug-likeness of lead compounds may be enhanced during the optimization process. Moreover, if a prospective therapeutic candidate shows excellent pharmacokinetics and toxicological characteristics compared to regular clinically accessible drugs, researchers should refrain from discarding the chemical, as it may prove to be a more effective alternative following preclinical and clinical trials [30, 229]. For instance, Osimertinib, a 3rd-generation epidermal growth factor receptor (EGFR) tyrosine kinase inhibitor (TKI), was developed to address drug-resistance variants that diminish the effectiveness of earlier-generation tyrosine kinase inhibitors, e.g., erlotinib and gefitinib, thereby enhancing overall pharmacokinetics and drug-likeness [230]. Its improved pharmacokinetics, characterized by increased brain penetration and reduced toxicity, has resulted in its endorsement as an excellent choice for the therapy of non-small-cell lung cancer [231, 232]. In another example, researchers have demonstrated that conventional liraglutide, a glucagon-like peptide-1 (GLP-1) receptor agonist, is effective for treating type 2 diabetes and obesity. This ligand initially exhibited significant adverse effects due to its substantial molecular size and inadequate oral bioavailability, hindering its involvement in preliminary pharmacological trials [233, 234]. However, researchers modified the compound by linking fatty acid chains to extend its half-life and reduce absorption, so enhancing its pharmacokinetic profile, which has shown to be effective in managing diabetes and facilitating weight loss. The redesigned fatty acid-modified liraglutide demonstrated excellent docking and binding selectivity, along with improved toxicological and pharmacokinetic characteristics compared to standard clinically accessible drugs [235–237].

### Accuracy limitations of ADMET models

The accuracy of traditional in-silico ADMET techniques for forecasting the pharmacokinetics and pharmacodynamics of drug candidates is a concern among the scientific community [93, 238]. Although in silico research relies on predictions, it is imperative for researchers to evaluate the tools to ensure precise study outcomes. Ametsar2 has been employed to forecast human ether-à-go-go-related gene (hERG) inhibitors, mutagenicity, and hepatoxicity, which raises apprehensions regarding the model’s reliability. Despite advancements in ADMET models integrated into software and web servers, further efforts are required to enhance the in vitro and in vivo datasets utilized for constructing these models [115, 133]. Recently, researchers have investigated this issue, contending that it is essential to compare the outcomes of different in silico methods to prevent erroneous toxicological and pharmacokinetics predictions, both false positives and false negatives [239–241]. Moreover, they proposed that several computational ADMET tools are constructed using distinct algorithms and models, necessitating a comparison of prediction outcomes to determine the most reliable research endpoint [218, 221, 242–244]. For example, researchers have demonstrated a more potent ligand and evaluated new PIM1 kinase inhibitors using molecular docking and in silico ADMET analysis. Thirty triazolo [4,3-b] pyridazin-3-yl-quinoline conjugates were examined for their ability to inhibit PIM1 kinase aggregation. Molecular docking between the ligand, viz., compound 25, and the PIM1 kinase active site was utilized to predict binding interactions, and the results showed impressive molecular recognition. Furthermore, the suggested inhibitor possessed favourable ADMET properties, e.g., increased oral bioavailability, low toxicity, and efficient cell membrane permeability, which highlighted its potential as a strong PIM1 kinase inhibitor, according to the in silico ADMET analysis of pharmacokinetics and toxicity investigations [245, 246].

## Experimental validation and integration

Upon attaining a comprehensive understanding of ADMET modelling and prediction, this section evaluates the experimental validation and integration. Artificial intelligence and computational methodologies are advantageous techniques for identifying novel chemical entities for illness treatment or management [116, 247–249]. The artificial intelligence and in silico investigations have revealed active compounds that impede target receptor activity. Recent research, including Acharya’s supercomputer-driven ensemble docking and replica-exchange molecular dynamics investigations, resulted in the identification of approximately 70 development candidates for the treatment of Covid-19 [45, 103, 250]. Nevertheless, it is crucial to acknowledge that artificial intelligence and computational methodologies by themselves do not ensure effectiveness in the absence of rigorous experimental and translational validation. This section examines extensive research employing experimental approaches to facilitate the validation of artificial intelligence and in silico-identified lead candidates and evaluates works in which computational methodologies underpin empirically derived development candidates.

### The experimental validation of artificial intelligence and computationally identified drug candidates

Artificial intelligence and computational methodologies are progressively employed to investigate molecular targets for lead discovery, with numerous works using experimental results to corroborate artificial intelligence and computationally identified drugs. Studies exemplified in Table 1 have attracted significant interest and demonstrate the efficacy of artificial intelligence and computational methodologies in drug discovery.

**Table 1:** The drug candidates identified using artificial intelligence and computational methodologies, subsequently verified through experimental techniques

No	Ligands or drug candidates	Software	Pathology	Ref
1	Cannabinoid receptors	V-SYNTHES	Neurodegenerative disease	[69]
2	Discoidin Domain Receptor 1 (DDR1) inhibitors	GENTRL	Cancer	[251]
3	(±)−2-Cyclohexyl-5-methoxy-2H-chromene	Cresset’s Spark	Neuroprotective	[252]
4	Bis-1,2,3-Triazoles	Flare™	Anti-Proliferative	[253]
5	Eltrombopag, folic acid, fexofenadine, indapamide, montelukast, and Ticagrelor	Cresset Flare™	PD-1 and PD-L1 inhibitors	[254]
6	(chalcone derivative (B-4), pyrazoline–carbothioamide (B-9), and pyrazoline–thiazole hybrids (BH1-7))	Schrödinger v2022-4	Cancer	[255]
7	BarVac vaccine	ClusPro, Cresset's Flare™	Cat scratch disease	[256]
8	Trichostatin A and CYS-145 thiol	CovalentDock	COVID 19	[104]
9	N3	GLIDE v8.2	COVID 19	[88]
10	Nafamostat, Camostat, and Gabexate	MOE dock Auto dock Vina	COVID 19	[250]
11	2-NMPA derivatives	Schrödinger Maestro v13.1	Osteoporosis	[257]
12	Carmofur	CDOCKER BIOVIA	SARS-CoV-2	[258]
13	N-[benzyl(4-phenylbutyl) carbamoyl] amino acid	CDOCKER BIOVIA	Sclerosis	[259]
14	1,5-disubstituted α-amino tetrazole derivatives	ChemAxon suite, Babel	Anti-inflammatory and immune disorders	[260]
15	PZM21	DOCK v3.6	OPOID ANALGESIC	[261]
16	(S)−1-((2-((4,4-bis (ethoxy methyl)cyclohexyl)oxy) benzyl)(methyl)amino)−3-(methylamino) propan-2-ol	Schrödinger v11.8	Colorectal cancer (CARM1 inhibitors)	[261]
17	4-(4-methyl-4H-1,2,4-triazol-3-yl) piperidine derivatives	Schrödinger Maestro v13.1	Anti-cancer	[262]
18	Amentoflavone derivatives	Cytoscape v3.8.2	COVID 19	[263]
19	triazolo[4,3-b]pyridazin-3-yl-quinolinederivatives	AutoDock v4.2, Molegro Virtual Docker v6.0, Gromacs v2018.1	Cancer (PIM1 kinase inhibitor)	[264]
20	Glabridin, 5′-formylglabridin, R- glabridin, 5′-prenyl glabridin, glabri din dimer and 3′-hydroxy-4′methoxyglabridin	Autodock vina GROMACS v5.1.2	Anti-melanogenic	[265]
21	Benzyloxychalcone	AutoDock Tools v1.5.6rc3 GROMACS v2022.4	Alzheimer’s disease	[266]
22	Diclofenac and its derivative	PyRx v0.8	Antipyretic, anti-inflammatory	[101]
23	Furo[2,3-b]indol-3a-ol	GuassView v6.0 Schrödinger’s Maestro	Cancer	[190]
24	Indeno [1,2-b] pyrrol-4 (1H)-one	Schrödinger Maestro	SARS-CoV-2	[267]
25	Cytidine derivatives	AutoDock v4.2.6, Swiss-Pdb v4.1.0, Python v3.8.2 Discovery Studio v4.1	SARS-CoV-2 RdRp	[92]
26	1,2,4-triazine-3(2H)-one derivatives	AutoDock GROMACS v2019.3	Breast cancer	[268]
27	2,4-dimethyl benzene-1,3-diol	AutoDock GROMACS v2019.3	Cancer	[243]
28	1,2,4 thiadiazole derivatives	Maestro V2.0	Antiproliferative	[269]
29	Ethyl 2-amino-6-methyl-4,5,6,7-tetrahydrothieno[2,3-c] pyridine-3-carboxylate	MOE v2015	Cancer	[270]
30	2-(2-butyl-1,3-dioxoisoindoline-5-carboxamido) − 4,5- dimethoxy benzoic acid (Cpd39)	GLIDE, GOLD, Autodock Vina	Prostate cancer	[96]

Recent research has shown the enhanced success rates associated with modular synthon-based strategies in the identification of high-affinity ligands across extensive chemical spaces (Table 1) [69]. The implementation of virtual synthon hierarchical enumeration screening in the context of cannabinoid receptors, e.g., *CB*_1_ and *CB*_2_, facilitated the effective investigation of the 11-billion-compound Enamine REAL space library. The synthon-based approach resulted in an experimental hit rate of approximately 30% for novel cannabinoid antagonists. The experimental validation conducted through radioligand binding assays substantiated the identification of around 15 submicromolar ligands. Following this, lead optimization efforts generated a candidate exhibiting an excellent inhibitory constant (*K*_i_) of approximately 1.0 nM.

Researchers have also shown that data-driven deep learning approaches markedly enhance both the hit rate and the structural diversity of novel kinase inhibitors (Table 1) [271]. A representative instance is the identification of nanomolar inhibitors for the discoidin domain receptor 1 kinase, a target that has historically posed difficulties for conventional docking methodologies owing to its intricate conformational plasticity. Through the use of a Generative Tensorial Reinforcement Learning (GENTRL) framework, researchers successfully conducted de novo design and validation of potent candidates within approximately 50 days. The experimental validation conducted via enzymatic inhibition assays established that the principal compound generated by artificial intelligence exhibited a half-maximal inhibitory concentration (*IC*_50_) value of 11 nM. Furthermore, subsequent cellular assays revealed a high degree of selectivity and advantageous pharmacokinetic characteristics.

Research studies have also shown covalent-based docking and subsequent experiments as a viable approach for identifying ligand inhibitors. A virtual screening effort was conducted, resulting in the identification of approximately 15 covalent Janus kinase 3 (JAK3) inhibitors [272–274]. In the experimental studies, the most effective docking molecules, viz., Cyanocrylamides 31 and 33, were evaluated in vitro against JAK3 and another three JAK-family kinases using complete dose–response curves. The findings revealed that the two covalent inhibitors exclusively targeted the JAK3 enzyme, with half-maximal inhibitory concentration values of approximately 40 nM and 90 nM, respectively [275]. This indicates that the covalent attachment of the molecular probes with the cysteine residue is essential for the probe’s inhibitory efficacy.

The researchers have also identified PZM21 as a new agonist of the µ-opiod receptor, potentially more effective than morphine for pain management [276, 277]. Through computational docking and 3.0 µs molecular dynamics simulations, they showed the robustness of the docked pose, retaining crucial hydrogen bonds and ionic interactions. In experimental studies, an irreversible variant, PZM29, was synthesized to validate PZM21’s efficacy as an opioid agonist. The optimized structure of PZM21 shows a strong binding affinity, e.g., *K*_i_ = 1.1 nM, and functions as a *G*_i_ biased agonist, activating the *G*_i_ protein pathway while minimizing β-arrestin-2 recruitment, thus reducing opioid-related side effects, viz., respiratory depression. The in vivo hotplate assay showed a dose-dependent analgesic effect of PZM21, achieving 87% efficacy after 180 min, outperforming morphine. PZM21 showed selectivity for the µ-opioid receptor without significant activity against other opioid or nociception receptors, further reducing potential side effects. The GTPγS assay for PZM21 revealed a half-maximal effective concentration (*EC*_50_) value of 8.20 ± 0.09 for opioid receptor signalling and 7.55 ± 0.15 for receptor internalization, indicating differential potency in activating signalling versus uptake. Additionally, the respiratory depression assessment on an unrestrained mouse model at 40 mg/kg concentration demonstrated that PZM21 had minimal side effects [277–279].

Researchers have also conducted an in silico structure-based screening of an extensive chemical library and subsequent experiments to identify potential inhibitors of SARS-CoV-2 Mpro [104]. The use of computational methodologies, viz., virtual screening and molecular docking of approximately 8,800 compounds, has led to the identification of Trichostatin A as a significant antagonist of SARS-CoV-2 Mpro. The in silico analysis revealed that Trichostatin A exhibits stable binding to the catalytic site, establishing hydrogen bonds with LEU-141 and HIS-163, and initiating a covalent thia-Michael addition with CYS-145. The stability was corroborated through free energy calculations and molecular dynamics simulations, which directly facilitated the experimental validation of Trichostatin A’s antiviral activity. In the experimental studies, the half-maximal effective concentration of Trichostatin A for suppressing SARS-CoV-2 proliferation was between 1.4 to 2.8 µM, significantly below its 50% cytotoxic concentration (*CC*_50_) of 75.7 µM [280, 281]. Moreover, researchers disclosed that Trichostatin A disrupted the phase of the SARS-CoV-2 proliferative cycle. Despite this accelerated metabolism, the principal metabolite of Trichostatin A exhibits histone deacetylases inhibitory activity. The half-maximal effective concentration value of Trichostatin A against SARS-CoV-2, as ascertained by plaque reduction assay, is approximately 1.5 μM, which is below the maximum plasma concentration (*C*_max_). The development and validation of Trichostatin A as a potent anti-SARS-CoV-2 drug molecule demonstrated the efficacy of the structure-based screening tools, with in vitro enzyme inhibition and antiviral tests, in identifying SARS-CoV-2 Mpro inhibitors from an extensive chemical library [104, 280, 281].

The research has also shown a mechanism-driven peptidomimetic antagonist, viz., N3, by computer-driven drug discovery and subsequent experiments. The researchers constructed the three-dimensional tertiary structure of SARS-CoV-2 Mpro and employed Glide software as a docking tool to target the receptor. The in silico investigation demonstrated that N3 might occupy the active site of the receptor target. In the empirical studies, an experimental kinetic assay for N3 was conducted to evaluate the inhibitor’s effectiveness, demonstrating that the Michael-acceptor molecule acts as an irreversible time-dependent inhibitor. Moreover, the researchers showed the crystal structure of Mpro co-crystallized with the antagonist. Additionally, the N3 exhibited inhibitory effects on SARS-CoV-2, with individual half-maximal effective concentration values of approximately 16.7 μM. The established half-maximal inhibitory concentration of N3 was also around 16.8 μM for Mpro. However, N3 exhibits an effective suppression of SARS-CoV-2 Mpro, rendering the conventional assessment of *K*_i_ and *K*_3_ impractical. Nevertheless, the *K*_obs_/[I] was employed to assess the suppression, during instances of fast inactivation, serving as an estimate of the pseudo-second-order rate constant, viz., *K*_3_/*K*_i_. The calculated value for *K*_obs_/[I] of N3 for SARS-CoV-2 Mpro was 11,200 ± 870 M^−1^ s^−1^, indicating that the Michael acceptor significantly hindered the enzyme [233, 282, 283].

Through the use of Gabexate, Camostat, and Nafamostat as repurposed scaffolds, researchers further conducted structure-based docking, molecular dynamics simulations, and subsequent experimental assays to identify novel inhibitors of TMPRSS2, utilizing these established drugs as initial benchmarks. The identified serine protease inhibitors established a foundational framework for the subsequent identification of the hits discussed in this investigation. Pharmacophore-based modelling was employed to evaluate a library of drugs possessing analogous active characteristics [233, 282, 283]. Following the identification of 20,000 lead-like pharmacophoric molecules, approximately 300 were chosen based on intermolecular hydrogen bonding with the target receptor, promiscuity, binding strength, and binding posture. In the experimental studies, biochemical assays were performed to assess the inhibitory effects of these compounds. Four drugs were experimentally identified as efficient inhibitors of TMPRSS2: NCGC00522442 (UK-1), NCG00421880, NCG00386945, and Ortamixaban [250, 283, 284]. In the SARS-CoV-2 pseudotyped entry assay, only Ortamixaban and NCGC00386945 demonstrated around 50% efficacy. Moreover, Otamixaban, with a half-maximal inhibitory concentration value of 0.62 μM, and NCGC00421880, with a half-maximal inhibitory concentration value of 0.88 μM, exhibit significant TMPRSS2 inhibitory activity. The selective inhibitor of urokinase, UK-1, possessed a half-maximal inhibitory concentration value of 2.20 μM, achieving complete inhibition. Furthermore, the fluorescence interference and inactivity in viral entry assays of UK-1 demonstrated inhibition with 50 − 70% efficacy [250, 285, 286].

### The computational evaluation of experimentally identified drug candidates

Computing methodologies are essential for analyzing the molecular processes of drugs identified by laboratory experiments. They include molecular and atomistic specifics regarding inhibition, binding interactions, and binding types. Table 2 delineates instances of the findings, and retrospective research are examined to offer a concise overview.

**Table 2:** The drug candidates identified using experimental procedures and subsequently evaluated using computational methodologies

No	Ligands or drug candidates	Software	Pathology	Ref
1	MDM2 binders	LiGaMD	Cancer	[287]
2	Ganoderic acid A	Maestro v9.6 GLIDE	Cancer	[288]
3	Nirmatrelvir	AutoDock	COVID-19	[289]
4	Phloroglucinol	AutoDock	Type-2 Diabetes	[290]
5	2‐(2‐(2‐Hydroxy‐5‐((p‐tolylthio)methyl)benzylidene)hydrazineyl)‐ 5‐(2‐(4‐methoxyphenyl)hydrazineylidene)thiazol‐4(5H)‐one (Compound 6 d)	MOE v2015.10	Antibacterial agent	[291]
6	Hydrazonothiazolines derivatives (compound 3 d)	MOE v2014.09	Antimicrobial (urease inhibitor)	[292]
7	1,3 diphenylurea derived Schiff bases (compound 5 h)	MOE v2022.02	Anti-diabetes (α-glucosidase inhibitors)	[293]
8	Thiadiazol derivatives ((compound 22)	MOE v2019 GROMACS v2021	Cancer (VEGFR-2 inhibitors)	[294]
9	Azoles derivative (compound 5 h)	MzDOCK v2.9	Antimicrobial agent	[295]
10	Pyrazole-phthalazine hybrids	PyRx AutoDock Vina GROMACS	Anti-diabetes (α-glucosidase inhibitors)	[296]
11	3-azolyl-4-chromanone phenylhydrazones	AutoDock vina	Antifungal	[297]
12	1-benzyl-indole hybrid thiosemicarbazones	Schrödinger Maestro glide	Tyrosinase inhibitors	[298]
13	Imidazole derivatives (compound AA6)	AutoDock v4.2	Anti-Inflammatory Activity	[299]
14	Chromone-peptidyl hybrids	Swiss-Model, ProMod DS visualiser	Antileishmanial agents	[300]
15	2-((3-(benzo[d]thiazol-2-yl)quinolin-2-yl)thio)-N-(4-fluorophenyl) acetamide	Schrödinger’s Maestro and Desmond	Anti-diabetes (α-glucosidase inhibitors)	[301]
16	Carnosic acid	Discovery Studio v4.5	Anti-Inflammatory	[302]
17	Dihydroquercetin, kaempferol, quercetin	AutoDock	Antibacterial	[303]
18	2-[5-(4-Chlorophenyl)−1,3-thiazol-2-yl]−1-acetylhydrazine	AutoDock	Breast cancer	[304]
19	Anthocyanins	Autodock v4.2, Autogrid v4.2	Porcine pancreatic α-amylas inhibitor	[305]
20	Quercetin and Daidzin	AutoDock Vina	Anticancer Agents	[306]
21	Isokaempferide	Schrödinger	Anti-cancer	[307]
22	Mulberrofuran M and (B) quercetin 3-(6″-caffeoylsophoroside)	AutoDock Vina	Antidiabetic Agent	[308]
23	Dicoumarols	AutoDock Vina	*Mycobacterium tuberculosis*	[181]
24	Luteolin, Apigenin, Caffeic acid, D-Cinnamic acid, Protocatechuic acid, Catechin, Ferulic acid, Gentisic acid, Jatrorrhizine	AutoDock Vina v1.1.2	Cancer	[309]
25	8-(3-(1H-imidazol-1-ylpropylamino)−3-methyl-7-((naphthalen-3-yl)methyl)−1H-purine-2, 6 (3H,7H)-dione (compound 5)	AutoDock v4.2	Cancer	[310]

Recent studies have investigated Gaussian accelerated Molecular Dynamics (GaMD) simulations (Table 2) [287], which functions as a comprehensive validation instrument in the realm of computational drug design, adeptly accommodating protein flexibility to assess binding thermodynamics and kinetics. The Ligand Gaussian accelerated Molecular Dynamics (LiGaMD) variant, including triple boosts to enhance substrate dissociation, rebinding, and conformational alterations, has been effectively implemented in the context of the MDM2 oncology protein. Through the simulation of interactions between MDM2 and small molecules, e.g., Nutlin 3, as well as highly flexible peptides, viz., PMI and p53, the ligand Gaussian accelerated molecular dynamics effectively aligns with experimental observations regarding binding free energies and kinetic rates.

Researchers have also examined the potential application of experimentally tested dicoumarols as inhibitors of the Mur B enzyme inhibitors in *Mycobacterium tuberculosis* through computational methodologies (Table 2) [181, 311]. Additionally, the biological activity of dicoumarols derivatives were tested against *Staphylococcus aureus* Mur B. In the computational studies, molecular docking revealed that dicoumarols successfully dock to the binding site of the Mur B molecule. All dicoumarol derivatives exhibited substantial binding energies with the Mur B protein, ranging from −11.5 to −7.0 kcal/mol. This suggests that they hinder the enzyme’s function, which is required for bacterial cell wall formation. The molecular dynamics simulations elucidates the temporal robustness of the dicoumarol-Mur B complex. The molecular mechanics–Poisson-Boltzmann surface-area decomposition analysis was performed to assess the ligand–protein complex’s stability [312, 313]. The compound 6d exhibited solvent-accessible surface energies of –5.61 ± 0.24 kcal/mol and electrostatic energies of –5.59 ± 2.63 kcal/mol, indicating a heightened binding affinity of the ligand to the protein. The biological efficacy of dicoumarol derivatives has been evaluated against the *Staphylococcus aureus* Mur B utilizing logarithmic (Log-) minimum inhibitory concentration (MIC) values. The quantitative structure–activity relationship model aims to correlate the biological activity of dicoumarol derivatives, in relation to the logarithmic-minimum inhibitory concentration, with their molecular characteristics. The quantitative structure–activity relationship models suggested that the four most promising dicoumarols are active against *Staphylococcus aureus* Mur B. The modelling of ADMET properties disclosed that dicoumarols have excellent pharmacokinetic characteristics. This indicates that they may be well absorbed in the body and possess suitable distribution properties, rendering them appealing therapeutic candidates [312, 313].

Researchers have also investigated the binding kinetics and antagonist efficacy of phloroglucinol on the α-glucosidase enzyme by a synergistic enzyme-kinetic assay and computational methodologies (Table 2). A double-reciprocal Lineweaver Burk plot assay was utilized to examine the kinetics of non-competitive suppression of α-glucosidase activity, revealing that phloroglucinol inhibited α-glucosidase non-competitively, resulting in a significant reduction in apparent *V*_max_ while maintaining a consistent *K*_m_ value of 0.76 ± 0.07 mM. The phloroglucinol is likely associated with α-glucosidase at a specific inhibition site near to the active site, rather than inside the active site itself. However, the conventional secondary re-plot was parabolic instead of linear, indicating non-competitive inhibition with several inhibitory sites [290, 301]. In the computational studies, molecular docking reveals a binding energy of –7.48 kcal/mol, showing that phloroglucinol is mostly sequestered inside the α-glucosidase binding pocket. The key binding residues were identified using molecular docking with AutoDock Vina v1.1.2 and a homology model based on *S. cerevisiae* isomaltase, exhibiting 72% sequence identity [294, 305]. The optimal docking score exhibits a binding energy of –7.48 kcal/mol. The molecular dynamics simulations validated that ASP68 and ARG312 showed stable binding via the formation of hydrogen bonds with phloroglucinol [290, 301, 312, 314].

The researchers have also performed studies utilizing experimental assays and in silico methodologies to investigate the efficacy and binding kinetics of Teicoplanin with the major protease of SARS-CoV-2. They repurposed multiple approved small molecules against the enzyme target through experimentation, identifying Teicoplanin as the most potent inhibitor. The experimental procedures utilized included protease activity assays, fluorescence quenching, surface plasmon resonance, and size exclusion chromatography. The research showed that Teicoplanin is the most effective, with a half-maximal inhibitory concentration value of approximately 1.5 μM. Moreover, Teicoplanin has a significant affinity for the 3-chymotrypsin-like protease (3CL^Pro^), as shown by an estimated Stern–Volmer quenching constant of 2.5 × 10^5^ L·mol^−1^. Teicoplanin and 3-chymotrypsin-like protease have favourable interactions as demonstrated by surface plasmon resonance, generating a *K*_D_ of around 1.6 mM [30, 126]. This finding elucidates the Teicoplanin’s mechanism of action as a potential anti-Covid-19 therapy. In the computational studies, molecular docking of Teicoplanin revealed that the molecule fits accurately into the receptor’s active site, engaging with the His41 and Cys145 catalytic dyad of M^pro^. The docking score showed a robust binding energy of approximately –7.0 kcal/mol, verifying the compound’s pharmaceutical-like efficacy. It establishes hydrophobic interactions with Asp-187 and Glu-166, in addition to hydrogen and halogen bonds, therefore stabilizing the inhibitor binding pocket [315, 316].

## Challenges and potential solutions

Following a thorough comprehension of experimental validation and integration, this section investigates the challenges and possible solutions in drug discovery.

### Continued expansion of easily accessible chemical spaces

The advancement of effective methodologies for the screening of vast chemical spaces is crucial for the field of drug discovery, significantly improving the diversity and quality of the hits and leads that are identified. The xREAL extension of Enamine REAL space, e.g., designed for V-Synthes screening, presently encompasses approximately 170 billion compounds and possesses the capacity to expand to around 10^15^ compounds. This expansion can be realized through the use of a comprehensive set of building blocks, exemplified by the approximately 700 million units provided by MADE building blocks [93, 229, 238]. Moreover, the integration of more complex scaffolds, e.g., four-component and five-component variants, in conjunction with novel click-like chemistries, will promote this advancement. In order to assess the practical applicability of the MADE-enhanced REAL space and various corporate or private chemical libraries, it is imperative to conduct real-world tests to evaluate their synthesizability and overall effectiveness. Furthermore, there exists a potential to develop specialized ultra-large libraries that concentrate on critical scaffolds, which are often inadequately represented in broad-purpose synthetically-accessible environments [93, 238]. For instance, recent screenings of a chemical library comprising approximately 80 million readily synthesizable tetrahydropyridines have led to the identification of strong agonists for the 5-HT2A receptor.

The expansion in the scale and variety of the on-demand chemical domain is substantially enhanced by the creation of novel, resilient processes intended for the click-based integration of building elements. This encompasses the conventional azide-alkyne cycloaddition click chemistry, which received the 2022 Nobel Prize in Chemistry, as well as advanced click-like reaction, e.g., Sulfur(VI) fluoride exchange (SuFEx). The emergence of nickel-electrocatalyzed doubly decarboxylative cross-coupling methods signifies additional potential in this field. Furthermore, novel carbon–carbon generating processes that employ methyliminodiacetic acid boronates provide a foundation for *Csp*^2^*–Csp*^2^ couplings [317, 318]. Recent advancements have involved the use of tetramethyl N-methyliminodiacetic acid boronates in the stereospecific formation of *Csp*^3^*–C* bonds. When these reactions are applied in an iterative manner, they enable the development of extensive synthetically-accessible virtual libraries, encompassing billions of varied molecules through the utilization of a limited set of building blocks. This concept is analogous to the automated formation of amino acids in peptide production, indicating that completely computerized systems may be utilized to generate libraries of pharmaceutical-like molecules on request through the combination of several thousand varied building elements. Although these robotic systems are currently in operation, there are persistent challenges associated with scaling the generation of thousands of specific building elements, which has been recognized as a considerable bottleneck in the overall process.

The advancement of resilient generative molecular spaces is also greatly enhanced by innovative computing techniques in the field of synthetic chemistry. The methodologies encompass sophisticated predictions of iterative reaction sequences and the assessment of synthetic routes through the use of deep learning-based retrosynthetic analysis [319–321]. Within the domain of generative models, the capacity to forecast synthesizability can be amalgamated with evaluations of potency and other pertinent characteristics, thus augmenting the automation in chemical design. An important use of this is the integration of generative adversarial networks with reinforcement learning (GAN-RL), which has been utilized to forecast the synthesizability, uniqueness, and biological behaviour of diverse compounds. This integration enables a repetitive process of computational enhancement, production, and laboratory evaluation of ligands. When applied within clearly delineated reaction sets and pharmacologically characterized target classes, these methodologies have effectively identified significant hits and leads, advancing toward the development of clinical candidates [322–324]. Nevertheless, the wider ramifications and possibilities of computerized molecular design and automated synthesis in the realm of drug development remain to be comprehensively understood.

### Inability of databases to represent “negative space”

Within the framework of biomolecular databases such as PDBbind, the concept of “negative space” also pertains to negative data, viz., it encompasses information regarding pairs of proteins and ligands that exhibit no binding affinity or demonstrate only minimal interaction [325, 326]. The insufficiency of a satisfactory representation of negative space constitutes a considerable obstacle for artificial intelligence and drug discovery for various reasons. For instance, the PDBbind is established to compile experimentally determined binding affinities for complexes that are already documented in the Protein Data Bank [327–331]. Given that the Protein Data Bank predominantly catalogues successful structural determinations of stable complexes, the database is inherently characterized by an abundance of positive binders. It is deficient in a balanced counter-set of “decoys”, i.e., molecules that exhibit characteristics suggesting they should bind, yet ultimately do no succeed in a laboratory context [329–331]. Moreover, when an artificial intelligence model is exclusively trained on instances of successful positive outcomes, it encounters difficulties in comprehending the limitations that delineates the characteristics of non-functional molecules. The models may exhibit elevated accuracy during testing due to the lack of challenge in differentiating genuine binders from near-miss inactives. In the absence of negative space, models frequently depend on the acquisition of straightforward correlations, e.g., molecular size or surface area, instead of engaging with the intricate physics of interactions [332, 333]. This reliance results in suboptimal performance when confronted with entirely novel chemical libraries. Furthermore, in the field of structural biology, the term negative space denotes the physical cavities or unoccupied volumes within a protein that are devoid of a ligand [334–337]. Databases, e.g., PDBbind, provide insights into the spatial arrangement of ligands; however, they frequently lack explicit delineation of the regions within a binding pocket that are unoccupied or deemed forbidden as a result of steric clashes [338–340]. Nevertheless, investigations have demonstrated that the development of a “negative image” of a binding site, i.e., conceptualizing the unoccupied cavity as a physical template, enhances the identification of novel drug candidates in comparison to conventional methodologies [338–342].

### Combining computational and experimental methods for complex drug targets

Contemporary computational methodologies have demonstrated potential through blind benchmarking and recent successful screenings for diverse targets. Nevertheless, there exist substantial categories of complex targets for which existing in silico screening methodologies may not suffice independently. Among the most challenging targets are those defined by cryptic or shallow binding pockets, necessitating that these pockets either become accessible or experience substantial conformation alterations, i.e., induced fit, to facilitate successful ligand binding. This challenge is especially pronounced when examining allosteric sites [343–345], as demonstrated in kinases and G protein-coupled receptors, along with protein–protein interactions within signalling cascades.

Computational methodologies in molecular dynamics and bioinformatics represent significant instruments for the identification and examination of allosteric and cryptic pockets; however, these methodologies frequently prove inadequate in facilitating ligand discovery within these intricate environments [346, 347]. Fragment-based drug discovery has developed into an effective approach for tackling the complexities associated with cryptic and shallow binding sites. This methodology commences with the experimental screening of small fragments to identify preliminary hits utilizing highly sensitive techniques, e.g., cryo-electron microscopy, X-ray crystallography, nuclear magnetic resonance, and surface plasmon resonance [348, 349]. These methodologies possess the capability to identify weak binding interactions generally within the concentration range of 10–100 µM. Preliminary screenings also employ fragments that have been altered with a reactive group to promote proximity-mediated covalent linking of weak-affinity ligands [77, 350, 351]. Nevertheless, the progression from preliminary fragment binders to fully developed strong-affinity ligands constitutes a considerable challenge within fragment-based drug discovery. This transformation necessitates a rigorous iterative process involving either the growth of fragments of the linkage of multiple fragments, which entails the design and synthesis of custom ligands that span several years [348, 352–354]. In the interim, structure-based virtual screening serves to enhance the computational refinement of fragments, ensuring their alignment with the experimentally determined conformation of the ligand-binding site. An economically efficient implementation of this virtual screening methodology transpires when fragment binders derive from synthetically-accessible spatial building elements or their closely related equivalents, thereby enabling uncomplicated development within an identical chemical domain [348, 355].

### Lack of application in deep learning

The advancement of the next generation of artificial intelligence-driven drug discovery is also transitioning from niche achievements to a focus on widespread applicability [356–358]. Although contemporary deep learning models have demonstrated excellent proficiency in particular tasks, e.g., predicting protein structures or identifying basic binders, they frequently encounter challenges when addressing the intricate complexities inherent in human biology [356–359]. The expansion of the applicability range necessitates several fundamental shifts. For example, deep learning models have to advance from small molecules to the design of biologics, peptides, and gene therapies while utilizing the same fundamental architectures [360, 361]. Moreover, rather than merely identifying a hit, the deep learning models are required to concurrently address toxicity, solubility, and metabolic stability, thereby ensuring that candidates are effective within a biological system, rather than solely in a simulated environment [362–364]. Furthermore, deep learning models should establish connections among cellular models, animal models, and human clinical outcomes to mitigate the elevated failure rate observed in Phase II and III trials. Overall, for artificial intelligence to transform the field of medicine, it is imperative that it evolves from a specialized instrument focused on particular protein families to a comprehensive system capable of addressing a wide array of disease platforms [365–367].

### Inconclusive benchmarking results of artificial intelligence

The benchmarking of Alphafold2 in virtual screening also generates inconsistent outcomes in two separate contexts: retrospective studies involving the redocking of known ligands and prospective studies aimed at the identification of new ligands [368, 369]. Although Alphafold2 demonstrates a high level of accuracy in predicting protein backbones, its efficacy in drug discovery exhibits considerable variability depending on the particular metric and target employed [180, 370]. For instance, in investigations where researchers attempt to redock ligands into their established binding sites utilizing Alphafold2 models, the outcomes frequently demonstrate a lower quality compared to experimental structures [371–373]. A comprehensive analysis of the PDBbind dataset revealed a success rate of approximately 20% for Alphafold2 models, in contrast to around 40% for experimental crystal structures [371–374]. Alphafold2 demonstrates proficiency in backbone prediction; however, it exhibits limitations in providing reliable fine structural details, e.g., side-chain orientations. The presence of these fine-grained inaccuracies frequently results in steric clashes that hinder the proper docking of ligands [371, 374, 375]. The Alphafold2 models generally depict the apo or unbound conformation of a protein. They frequently do not adequately represent the conformational alterations induced by the ligand, viz., the holo state, that are essential for achieving high-affinity binding [376, 377]. In contrast, certain prospective investigations have demonstrated that Alphafold2 predictions can be equally effective as experimental structures in the identification of new development candidates [373, 374]. In screenings involving millions of molecules against targets, e.g., the σ2 and 5-HT2A receptors, Alphafold2 models demonstrated hit rates, e.g., approximately 55%, that were nearly indistinguishable from those obtained using experimental structures [368, 373]. Researchers propose that Alphafold2 models have the potential to explore low-energy, alternative conformations of receptors that hold biological significance, yet diverge from the currently established crystal structures. This enables the identification of novel chemotypes that conventional structures overlook [368, 378–380]. In order to address these varied outcomes, researchers frequently employ particular refinement methodologies. For example, researchers have utilized the elimination of disordered loops, characterized by low predicted Local Distance Difference Test (pLDDT) scores, which has the potential to enhance the success rate of docking procedures. The researchers have also utilized molecular dynamics simulations or induced-fit docking to facilitate the Alphafold2 model in achieving a more drug-compatible conformation [75, 381].

### Limited accuracy of ADMET models

Although web-based ADMET predictors, e.g., SwissADME and pkCSM, provide rapid and accessible solutions, their accuracy is also frequently constrained by the breadth of their training datasets [382–384]. In instances where a hit compound is categorized within a novel chemical class that is inadequately represented in the server’s database, the resultant prediction transitions from interpolation to extrapolation, consequently resulting in “out-of-domain” errors. Several primary elements contribute to the complexity of these predictions [385–387]. For instance, a significant number of servers employ linear regression models or fundamental machine learning techniques that do not adequately consider the intricate biological cross-talk, e.g., multi-transporter interactions and non-linear metabolic pathways [97, 388]. Additionally, the data referred to as ground truth, utilized for the training of these servers, frequently originates from a range of laboratories that employ differing assay conditions, thereby introducing statistical bias into the resultant outputs of the software [388, 389]. Furthermore, numerous servers demonstrate proficiency in forecasting physical properties, e.g., solubility and lipophilicity; however, they encounter challenges when addressing dynamic outcomes, viz., the temporal variations in a drug’s concentration within a specific human organ or its long-term toxicological effects [97, 388, 389].

### Advancing a sustainable AI agenda within biopharmaceutical companies

Pharmaceutical companies have to prioritize governance structures that assess digital environmental implications to properly integrate sustainability into artificial intelligence strategy [390, 391]. Despite the potential of technology solutions, numerous obstacles to the sustainable adoption of artificial intelligence remains, e.g., organization opposition, challenges in integrating eco-friendly algorithms with current systems, and substantial initial investment costs. An incremental implementation plan, bolstered by executive endorsement and sustainability key performance indicators, is crucial for digital transformation initiatives [392–394]. Incentives such as internal carbon pricing stimulate innovation with environmental consciousness, potentially involving the incorporation of digital emissions into environmental, social, and governance (ESG) measurements and the advancement of green certification for cloud providers [395–397].

A feasible strategy is the implementation of “frugal artificial intelligence”, wherein algorithms are engineered for optimal performance with little resources. This approach has been exemplified by companies including DeepSeek in China and the French start-up company Nabla, which created lightweight artificial intelligence for medicinal purposes [398–401]. Moreover, pharmaceutical leaders, e.g., Sanofi and Novartis, have established standards by using artificial intelligence into eco-design and environmental monitoring strategies to minimize resource consumption. Furthermore, AstraZeneca’s pledge to plant 200 million trees by 2026, alongside the application of artificial intelligence to evaluate biodiversity, exemplifies their dedication [401, 402].

Biopharmaceutical companies are also contemplating the establishment of internal carbon pricing to enhance its comprehension of artificial intelligence’s environmental influence, thereby promoting innovation that is cognizant of ecological ramifications [402–405]. Policy advancements such as the Europe’s corporate sustainability reporting directive (CSRD) necessitate digital environmental transparency, while France’s REEN law advocates for energy-efficient digital methodologies. The biopharmaceutical companies have to not only adjust to these developments but also take the initiative in showcasing sustainable practices [403–405].

## Conclusions and outlook

The current review presents a prospective examination of the transformative effects of artificial intelligence and ultra-high performance computing methods on drug discovery. It underscores the transition from conventional computer-aided techniques to computer-driven strategies, accentuating the growing application of ultra-scale virtual screening methodologies and artificial intelligence-driven procedures for hit identification and lead optimization. The present review delineates existing research deficiencies, e.g., the constraints of conventional computer models in achieving predictive accuracy and the necessity for stringent experimental validation to improve the dependability of computational results. The proposed research goals encompass enhancing the robustness of computational models, integrating computational and experimental approaches, and tackling obstacles in targeting complicated protein–protein interactions and allosteric sites. By tackling these issues and utilizing upcoming technology, the current review article foresees a future in which artificial intelligence and ultra-high performance computing methods markedly improve the efficiency, precision, and accessibility of drug development procedures.

**Fig. 4:**
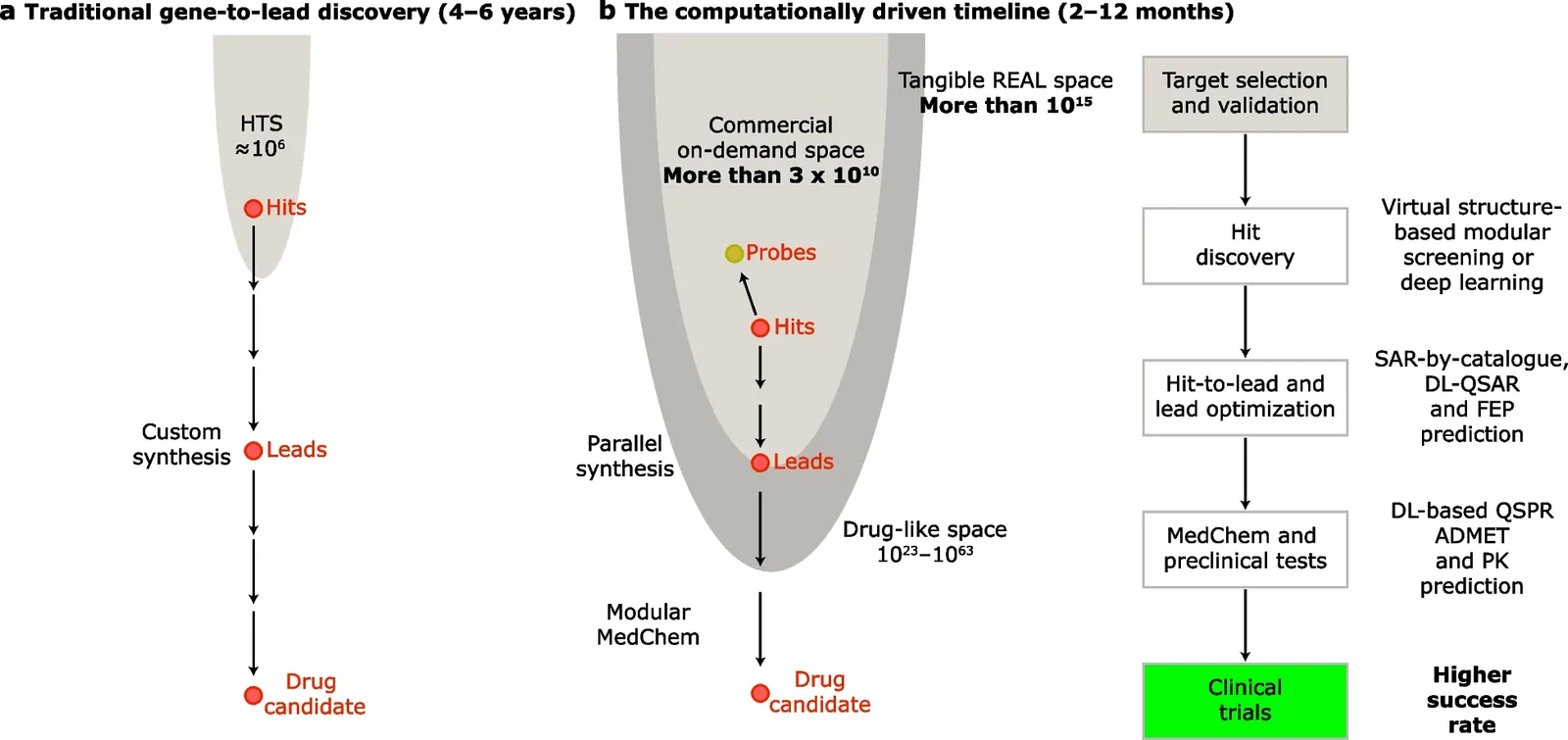
Computer-driven drug discovery. A schematic juxtaposition of the conventional high throughput screening combined with bespoke synthesis-driven discovery process with the computationally driven process. The latter uses structure-derived and artificial intelligence-based computational tools and on-demand or generative virtual molecular spaces to speed up drug discovery. The figure was created using Adobe Illustrator 2023

In the future, the increasing use of artificial intelligence and in silico methodologies in drug discovery and development is a significant shift from computer-aided to computer-driven approaches (Fig. 4). Gigascale in silico screening methodologies, e.g., structure-oriented and artificial intelligence-driven techniques, are increasingly used for hit identification [46, 171, 406]. During the hit-to-lead phase, potency prediction methodologies, viz., artificial intelligence-driven quantitative structure activity relationship and free energy perturbation, are employed to enhance ligand potency. Moreover, data-driven computational approaches are utilized for multiparameter optimization of lead series [43, 407]. Chemical spaces with over 10^10^ diverse compounds are anticipated to generate millions of initial active compounds, thousands of potent leads, and development candidates for nonclinical studies. However, to fully leverage this capability, traditional computing methods have to be improved for robustness and integration within the broader discovery pipeline.

Traditional computational models, however beneficial, also cannot assure the accuracy of all predictions; often, only 15–40% of candidate hits from virtual screening are validated experimentally [144, 172], with the precision of affinity predictions seldom surpassing a root-mean-square error of 1 kcal/mol. Contemporary computational models forecasting pharmacokinetics and ADMET characteristics face similar constraints, requiring rigorous experimental validation through in vitro and in vivo testing at every stage of the process [45, 83, 408]. Experimental validation generates data that enhances the model’s quality by augmenting their training datasets, particularly concerning ligand property predictions [45, 83, 408]. Deep learning-based quantitative structure–property relationship models benefit from the continued accumulation of data from functional organoids, organs-on-a-chip, or cell-permeability assays, to generate improved estimations of pharmacokinetics and ADMET properties without extensive in vivo experiments [130, 409, 410]. Creating pharmacokinetics and ADMET models using in vitro testing data from pertinent species, e.g., humans, rodents, and mouse, mitigate species variability, a significant obstacle in successful translational research. This establishes a beneficial process for enhancing computer models, facilitating compound selection for most drug discovery and development endpoints, and potentially eliminating the need for animal testing standards, as suggested by the FDA [411–413].

The integration of computational and experimental methodologies also facilitates exploration of the extensive chemical space in gene-to-lead procedures, creating different lead molecules with excellent selectivity, affinity, pharmacokinetics and ADMET characteristics. This minimizes compromises in multidimensional optimization for development candidates [244, 308, 414]. However, conventional data-intensive computational methodologies require comprehensive data management solutions for drug discovery, which are utilized in university drug discovery and development centres, and pharmaceutical companies. Nevertheless, computer-driven drug discovery possesses significant promise to reduce obstacles in the generation of compounds for various research avenues, e.g., personalized medicine, development candidates for rare diseases, multidimensional signalling, polypharmacology, and in vivo probes for novel targets [73, 415, 416].

Researchers have to also collaborate to establish protocols and guidelines for computer-driven drug discovery, building international partnerships and consortia [417–419]. The implementation of the Findable, Accessible, Interoperable, and Reusable (FAIR) standards is crucial for data sharing, [420, 421] improving the efficacy of deep learning and machine learning in a domain characterized by multiple unexplained processes and difficulties in reproducibility. The notion of “cloud labs” afford researchers remote access to standardized tools [358, 422–424]. The growing interest in data-driven drug discovery requires the incorporation of open scientific methods in companies, as numerous successful deep learning applications depend on data accessibility and open-source libraries. Despite the encouraging partnerships between academia and industry in open science, meticulous control of the hazards linked to the dissemination of research data and models is essential [424–426].

Recent studies have also analyzed the implementation of self-driving laboratories (SDLs) in drug discovery, [427–430] highlighting their transformational potential and the limitations that persist. Despite recent improvements in deep learning fostering optimism throughout the community, challenges such as the inaccuracy of artificial intelligence-generated ligand–protein binding data and the necessity for enhanced company transparency remain unresolved. Federated learning facilitates data confidentiality while enhancing collaboration. Anticipated progress in microfluidics technology and the establishment of BioFoundries are projected to improve self-driving laboratory implementation. The absence of cohesive feedback throughout several stages persists as a problem. Favourable regulatory changes, the significance of deep learning, and advancements in hardware indicate a promising environment for self-driving laboratory adoption in pharmaceutical science, potentially resulting in substantial enhancements in drug discovery.

Ultra-high performance computing has increasingly become fundamental to contemporary large-scale bioinformatics and drug discovery, significantly transforming how researchers simulate molecular behaviours and screen chemical libraries. The use of massively parallel architectures, e.g., graphic processing unit or tensor processing unit clusters, facilitates accelerated molecular dynamics simulations, frequently attaining speeds 10 to 100 times greater than conventional central processing unit-based solutions. For instance, the Highly Optimized Object-oriented Molecular Dynamics, blue edition, (HOOMD-blue) package employs graphic processing unit acceleration to effectively scale across numerous nodes [431], facilitating simulations of systems containing over 100 million particles. A significant advancement in this domain is the Anton supercomputer, which employs bespoke application-specific integrated circuit (ASIC) hardware to attain microsecond-scale molecular dynamics, facilitating the simulation of protein folding and drug-binding processes that exceed the capabilities of general-purpose machines. Furthermore, exascale computing platforms are transforming ultra-large virtual screening by enabling the evaluation of billions of compounds, viz., those in the Enamine REAL database, within days instead of years. Initiatives such as EXSCALATE, which utilizes extreme-scale computing to assess trillions of molecular interactions [432], demonstrate how ultra-high performance computing expedites drug discovery and improves researchers’ capacity to uncover novel inhibitors.

## Data Availability

Not applicable.
